# Lipidomic Remodeling in *Begonia grandis* Under Heat Stress

**DOI:** 10.3389/fpls.2022.843942

**Published:** 2022-02-17

**Authors:** Ai-Zhen Sun, Li-Sha Chen, Ming Tang, Juan-Hua Chen, Han Li, Xue-Qi Jin, Yin Yi, Fang-Qing Guo

**Affiliations:** ^1^National Key Laboratory of Plant Molecular Genetics, CAS Center for Excellence in Molecular Plant Sciences, Institute of Plant Physiology and Ecology, Chinese Academy of Sciences, Shanghai, China; ^2^Key Laboratory of State Forestry Administration on Biodiversity Conservation in Karst Mountainous Areas of Southwestern China, Guizhou Normal University, Guiyang, China; ^3^University of Chinese Academy of Sciences, Beijing, China; ^4^Key Laboratory of Plant Physiology and Developmental Regulation, School of Life Sciences, Guizhou Normal University, Guiyang, China

**Keywords:** heat stress, lipidomic analysis, triacylglycerols, phospholipids, lysolipids, sphingolipids, *Begonia grandis*

## Abstract

Characterization of the alterations in leaf lipidome in Begonia (*Begonia grandis Dry subsp. sinensis*) under heat stress will aid in understanding the mechanisms of stress adaptation to high-temperature stress often occurring during hot seasons at southern areas in China. The comparative lipidomic analysis was performed using leaves taken from Begonia plants exposed to ambient temperature or heat stress. The amounts of total lipids and major lipid classes, including monoacylglycerol (MG), diacylglycerol (DG), triacylglycerols (TG), and ethanolamine-, choline-, serine-, inositol glycerophospholipids (PE, PC, PS, PI) and the variations in the content of lipid molecular species, were analyzed and identified by tandem high-resolution mass spectrometry. Upon exposure to heat stress, a substantial increase in three different types of TG, including 18:0/16:0/16:0, 16:0/16:0/18:1, and 18:3/18:3/18:3, was detected, which marked the first stage of adaptation processes. Notably, the reduced accumulation of some phospholipids, including PI, PC, and phosphatidylglycerol (PG) was accompanied by an increased accumulation of PS, PE, and phosphatidic acid (PA) under heat stress. In contrast to the significant increase in the abundance of TG, all of the detected lysophospholipids and sphingolipids were dramatically reduced in the Begonia leaves exposed to heat stress, suggesting that a very dynamic and specified lipid remodeling process is highly coordinated and synchronized in adaptation to heat stress in Begonia plants.

## Introduction

As a consequence of global warming, high-temperature stress has been an adverse impact on almost all aspects of plant development, growth, reproduction, and yield and it has been assumed that the frequency and amplitude of hot waves during growing seasons for crop plants are expected to increase in the coming years (Baniwal et al., 2004; Wahid et al., 2007; Allakhverdiev et al., 2008; Ainsworth and Ort, 2010; Mittler et al., 2012; Li et al., 2019a). Begonia (*Begonia grandis*) belongs to the family Begoniaceae of annual ornamental plants that are commonly grown in city parks and as wild plants grown in fields and mountainous regions mainly due to the wide color options of flowers and adaptability to grow either in full sun or semi-shade worldwide. Due to global climate changes, the wax begonia plants were reported to often suffer from high-temperature stress during hot seasons (Lin et al., 2011). Over the last decades, extensive studies have been focusing on molecular mechanisms of basal and acquired heat tolerance for model plants, such as Arabidopsis and rice (Queitsch et al., 2000; Panchuk et al., 2002; Allakhverdiev et al., 2008; Mittler et al., 2012; Li et al., 2019a) whereas a few of investigations are involved in thermotolerance mechanisms of wild plants, such as plants in the family Begoniaceae.

Due to climate change in recent years, land plants are subjected to high-temperature stress with harmful effects on cellular membrane systems, such as an increase in molecular disorder and disintegration of lipid bilayers (Murata and Los, 1997; Los and Murata, 2004; Higashi and Saito, 2019). Generally, lipids play critical roles acting as metabolic, regulatory, and structural domains required for growth and development and in responses and adaptation to environmental stresses in plants (Welti et al., 2007; Okazaki and Saito, 2014; Hou et al., 2016). A wide range of lipid classes have been characterized as lipid signaling molecules including lysophospholipid, fatty acid, phosphatidic acid, inositol phosphate, diacylglycerol, oxylipin, sphingolipid, and *N-*acylethanolamine (Okazaki and Saito, 2014; Hou et al., 2016; Higashi and Saito, 2019). Several lines of evidence suggest that these signaling lipids are usually to be detected in small quantities in plant tissues, and upon on environmental stresses, these molecules can be rapidly synthesized from pre-existing membrane lipids or biosynthetic intermediates of membrane lipids (Okazaki and Saito, 2014; Hou et al., 2016). As the major constituents of biological membranes, lipids are determinant factors in controlling membrane fluidity and stability based on the lipid composition and fatty acid unsaturation levels that underpin the structure and function of cells (Zheng et al., 2011; Higashi and Saito, 2019). Accumulated data support that lipid remodeling is characterized as a marked physiological process for adaptation to heat stress in higher plants as plants need to maintain the stability of membrane bilayer and prevent lipid peroxidation under heat stress (Higashi and Saito, 2019). In addition to resulting in increase in peroxidation of unsaturated fatty acids, heat stress also causes the increasing frequency of membrane phase separation of non-bilayer-forming glycerolipids (Gounaris et al., 1983; Lee, 2000). Based on the studies on lipid remodeling, heat-induced significant reduction in lipid unsaturation levels by replacing the highly unsaturated lipids with less unsaturated ones has been characterized as a marked stress-responsive cascade across species, which is thought to be an adaptation mechanism to heat stress to prevent the phase transition of membranes from a bilayer to non-bilayer phase in plants. In Arabidopsis, the mutations of genes encoding plastidic fatty acid desaturase led to reductions in the number of glycerolipid fatty acid double bonds, resulting in enhancement in heat tolerance of the corresponding mutant plants, suggesting that changes in the proportion of glycerolipid composition in chloroplasts are dynamic in accordance with the stress strength and duration, which is required for plant acclimatization to heat stress (Kunst et al., 1989; Murakami et al., 2000; Routaboul et al., 2012). With respect to high-temperature stress, lipid remodeling has been extensively investigated in a variety of plant species including Arabidopsis (*Arabidopsis thaliana*; Burke et al., 2000; Chen et al., 2006; Higashi et al., 2015), rice (*Oryza sativa* var. *Japonica*; Navarro-Reig et al., 2019), wheat (*Triticum aestivum* L.; Narayanan et al., 2016a,b, 2018; Djanaguiraman et al., 2018, 2020), maize (*Zea mays* L.; Chen et al., 2010), soybean (*Glycine max* L.; Narayanan et al., 2020), tomato (*Solanum lycopersicum*; Spicher et al., 2016), tall fescue (*Festuca arundinacea*; Zhang et al., 2019), and creeping bentgrass (*Agrostis stolonifera*; Larkindale and Huang, 2004).

In this study, we aim at evaluating the leaf lipidome of Begonia to identify changes in lipid traits or species composition under heat stress that may provide deep insights into better understanding the acclimation and adaptation mechanisms to high-temperature stress since global warming becomes more and more challenging for terrestrial plants in recent years. We hypothesized that understanding the mechanisms of heat tolerance is critical to developing climate-resilient Begonia varieties.

## Materials and Methods

### Plant Materials and Growth Conditions

The seed of Begonia (*B*. *grandis Dry subsp. Sinensis*) was obtained from Dr. Yin Yi’s laboratory at school of life sciences, Guizhou Normal University, China. The seed of Begonia was stratified at 4°C for 7 d and then sown into peat soils (The Pindstrup Group, Denmark). The seeded pots were then transferred and grown in a phytotron under long-day conditions, 16 h of white light (80 μmol m^−2^ s^−1^) and 8 h of dark, with 60% relative air humidity at 22°C ± 1°C. The plants of Begonia were watered three times each week and fertilized weekly.

### Heat Treatment

Fully extended leaves were detached from 173-d-old plants of Begonia. The detached leaves were placed on plastic square Petri dishes with three-layer Whatman filter paper at the bottom immersed in 10 ml of deionized water. The plastic square Petri dishes containing the detached leaves were subsequently incubated at control (22°C for 2 h) or heat stress (45°C for 2 h) conditions in dark.

### Lipid Extraction

Following control or heat treatment, five biological samples of detached leaves for control or heat treatment were taken, respectively. Each sample of detached leaves (1 g FW) was quickly frozen in liquid nitrogen and stored at -80°C. Total lipids were extracted according to the protocol (Bligh and Dyer, 1959) with modifications. The frozen leaves were ground in liquid nitrogen, and the resulting tissue pellets were re-suspended in 2 ml de-mineralized water followed by the sequential addition of 2.4 ml precooled methanol and 8 ml of methyl tert-butyl ether with vigorous mixing after adding each solvent. Ultrasound was applied for 20 min at low temperature water bath, and the resulted sample was kept at room temperature for 30 min. Organic and water phases were separated by centrifugation at 14000 *g* for 15 min at 10°C. The upper organic phase was transferred into a glass vial, dried under a stream of nitrogen gas at 37°C, and stored at −20°C.

### Chromatography Conditions for Lipid Analysis

Dried lipid extracts were reconstituted in 2 ml 90% isopropanol/acetonitrile with vigorous mixing and centrifuged at 14000 *g* for 15 min, and finally, 3 μl of the resulting sample was injected for analysis. Reverse phase chromatography was selected for LC separation using CSH C18 column (1.7 μm, 2.1 mm × 100 mm, Waters). Mobile phases were consisted of (A): acetonitrile–water (6:4, v/v) with 0.1% formic acid and 0.1 mM ammonium formate; and (B): acetonitrile–isopropanol (1:9, v/v) with 0.1% formic acid and 0.1 mM ammonium formate. The initial mobile phase was 30% solvent B at a flow rate of 300 μl/min. It was held for 2 min and then linearly increased to 100% solvent B in 23 min, followed by equilibrating at 5% solvent B for 10 min.

### Mass Spectrometry Conditions for Lipid Analysis

Mass spectra were acquired by Q Exactive Plus mass spectrometer (Thermo Fisher Scientific) in positive and negative mode, respectively. ESI parameters were optimized and preset for all measurements as follows: Source temperature, 300°C; Capillary Temp, 350°C; the ion spray voltage was set at 3000 V; S-Lens RF Level was set at 50%, and the scan range of the instruments was set at *m/z* 200–1800. “Lipid Search” is a search engine for the identification of lipid species based on MS/MS data. Lipid Search contains more than 30 lipid classes and more than 1,500,000 fragment ions in the database. Both mass tolerance for precursor and fragment were set to 5 ppm.

### Data Analysis

Lipid search software (Thermo Fisher, Waltham, United States) was used for peak identification, retention time correction, and automatic integration pretreatment. After editing, the data matrices were imported into SIMCA-P 13.0 (Umetrics, Umea, Sweden), centered on the mean, and proportionally adjusted to Pareto variance. The multifactor analysis was then carried out. Partial least square discriminant analysis and orthogonal projection latent structure discriminant analysis (OPLS-DA) were used to analyze the data. The variable importance in the projection (VIP) value of the OPLS-DA model (VIP ≥ 1) and independent sample *t*-test (*p* < 0.05) were used to screen differential metabolites. Hierarchical cluster analysis was performed using pheatmap package of R language.

## Results and Discussion

### Heat Stress Induces Accumulation of Triacylglycerols (TG) in Leaves of *B. grandis*

**Figure 1:**
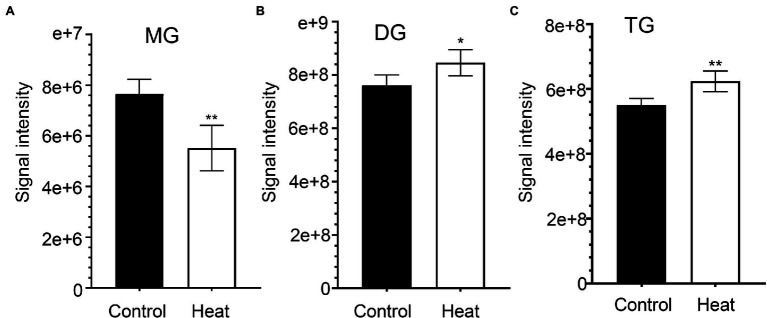
Effects of heat stress on accumulation of glycerolipids in leaves of *Begonia grandis*. **(A)** monoacylglycerol (MG); **(B)** diacylglycerol (DG); **(C)** triacylglycerol (TG). Fully extended leaves detached from 173-d-old plants of *B. grandis* were at control condition (22°C for 2 h) or challenged with heat stress (45°C for 2 h). Values shown are mean ± SD (*n* = 5). ^*^p < 0.05, ^**^p < 0.01, two-sided Student’s *t-*test.

**Figure 2:**
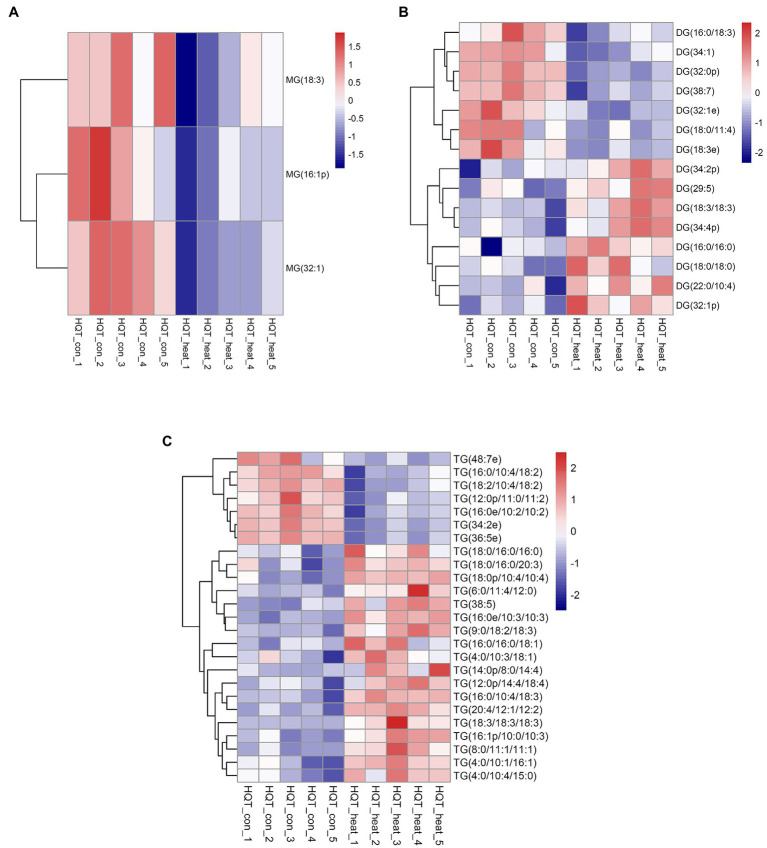
Heat map of profiles for the glycerolipid species identified in leaves of *B. grandis* under heat stress. Fully extended leaves detached from 173-d-old plants of *B. grandis* were at control condition (22°C for 2 h) or challenged with heat stress (45°C for 2 h). Each column represents an LC–MS measurement from five biological replicates of control condition (HQT_con_1 to HQT_con_5) or five biological replicates of heat treatment (HQT_heat_1 to HQT_heat_5). The glycerolipid species of **(A)** monoacylglycerol (MG), **(B)** diacylglycerol (DG), and **(C)** triacylglycerol (TG) measured from the control and heat treatment conditions were shown.

**Figure 3:**
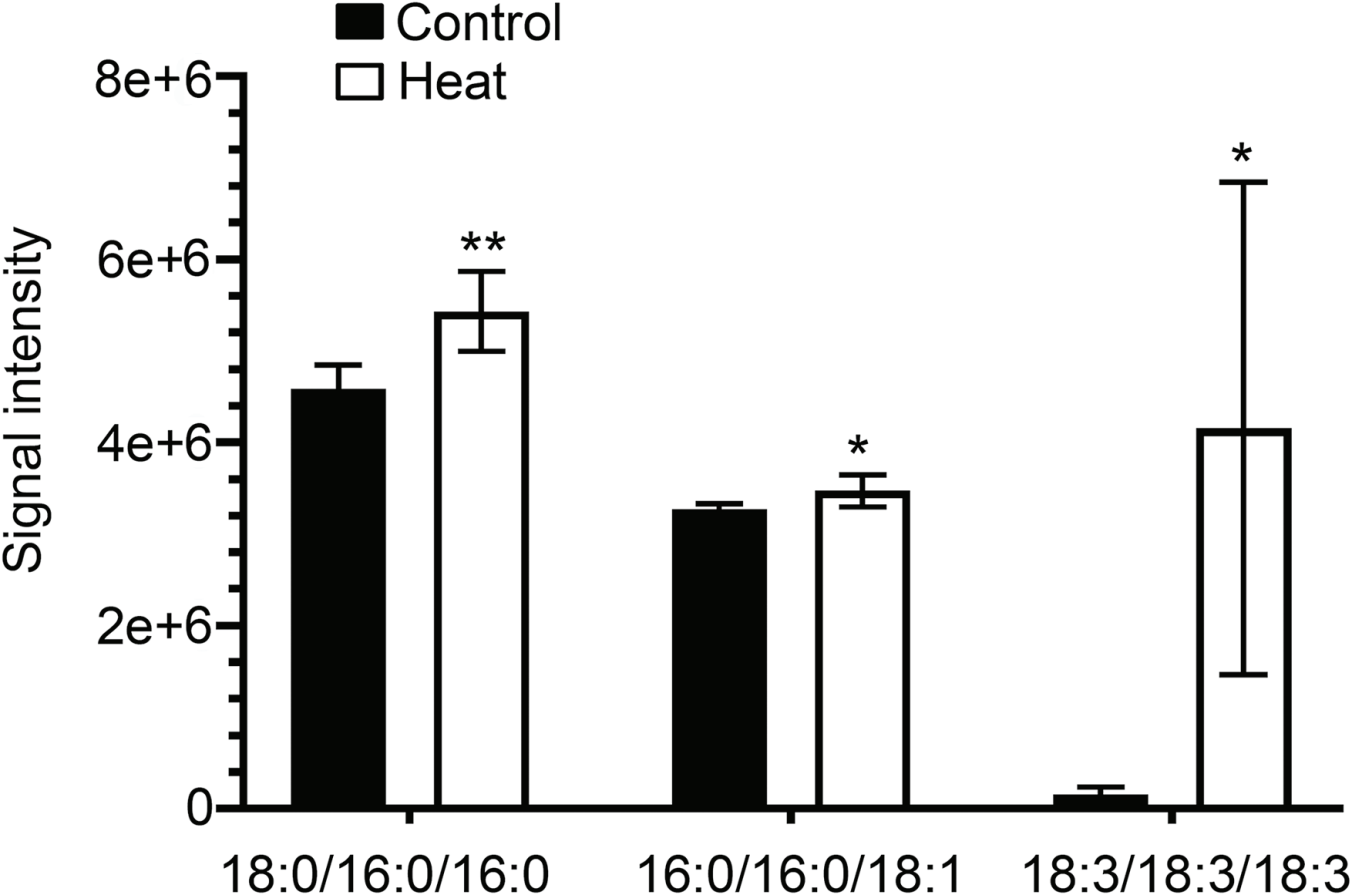
Changes in the levels of triacylglycerol lipid molecular species in response to heat stress. Fully extended leaves detached from 173-d-old plants of *B. grandis* were at control condition (22°C for 2 h) or challenged with heat stress (45°C for 2 h). Three different molecular species of triacylglycerol (TG) including 18:0/16:0/16:0, 16:0/16:0/18:1, and 18:3/18:3/18:3 in leaves of *B. grandis* were shown. Values shown are mean ± SD (*n* = 5). ^*^p < 0.05, ^**^p < 0.01, two-sided Student’s *t-*test.

Cellular membrane systems including plasma and chloroplast membranes are very sensitive and easy to be altered when subjected to adverse environmental conditions, such as high-temperature stress (Hou et al., 2016; Higashi and Saito, 2019). At high temperature, glycerolipids as the major constituents of membranes can be changed into a very dynamic remodeling process by adjusting the glycerolipid composition of the related membranes with keeping the integrity and optimal fluidity of the membrane systems (Higashi and Saito, 2019). The contents of diacylglycerols (DG) and triacylglycerols (TG) significantly increased in leaves of *B. grandis* in response to heat stress (45°C, 2 h) whereas the reduced content was measured for monoacylglycerols (MG; Figures 1A–C) with considerable variations among different glycerol lipid species (Figures 2A–C). Accumulated data from the published reports suggest that a cumulative effect of lipid remodeling has been marked by the decreases in unsaturation levels of membrane lipids occurring in response to high-temperature stress (Higashi et al., 2015; Higashi and Saito, 2019; Shiva et al., 2020). With respect to the levels of triacylglycerols containing less unsaturated fatty acids, such as oleic (18:1) and linoleic (18:2) acids, and/or saturated fatty acids, such as palmitic (16:0) acid, the heat-induced accumulations of TG (18:0/16:0/16:0), TG (16:0/16:0/18:1) were detected (Figure 3), suggesting that the significant increase in saturation index was driven by accumulation of these two TG species under heat stress in leaves of *B. grandis*. In addition to the increased levels of triacylglycerols containing less unsaturated and saturated fatty acids as mentioned above, a dramatic (26.7-fold) increase was detected in the level of TG containing highly unsaturated fatty acids, such as linolenic acid (18:3), in leaves of *B. grandis* when challenged with heat stress (Figure 3). Similar to our findings, this lipidomic remodeling patterns were also reported in Arabidopsis (Higashi et al., 2015; Mueller et al., 2015, 2017). Triacylglycerols are thought to act as buffers for the homeostasis of cellular acyl lipids with occurrence of a very dynamic recycling of 18:3 fatty acids in TGs (Hernandez et al., 2012). This suggests a possible sequestration of 18:3 acyl chains from plastidic and extraplastidic lipid species into TGs at high temperature. In combination with previous studies in other plant species, our findings suggest that fine-tuning the metabolic processes of glycerolipids is important for high-temperature stress acclimation in Begonia plants.

### Heat Stress Increases the Levels of PA, PE, and PS

**Figure 4:**
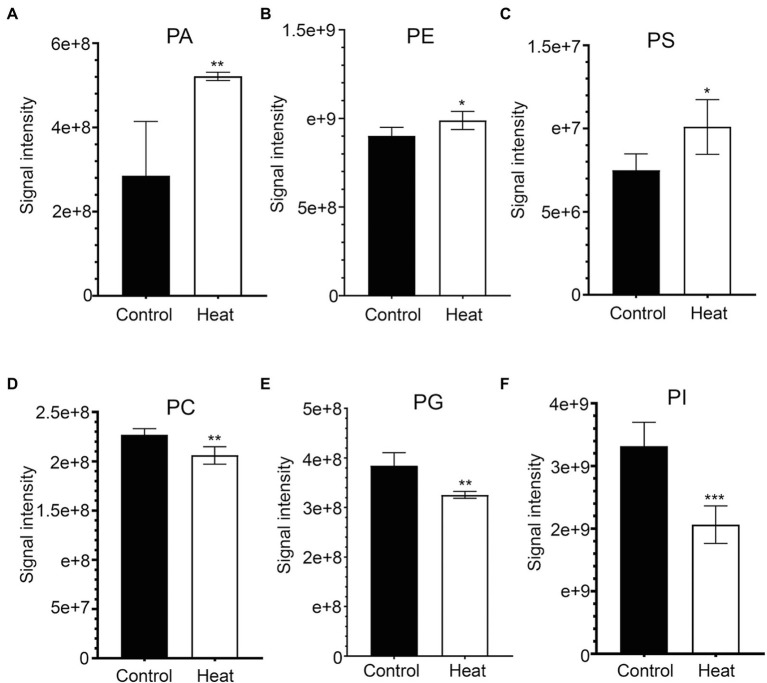
Heat-induced alterations in the levels of phospholipids in leaves of *B. grandis*. Fully extended leaves detached from 173-d-old plants of *B. grandis* were at control condition (22°C for 2 h) or challenged with heat stress (45°C for 2 h). **(A)** PA, phosphatidic acid; **(B)** PE, phosphatidylethanolamine; **(C)** PS, phosphatidylserine; **(D)** PC, phosphatidylcholine; **(E)** PG, phosphatidylglycerol; **(F)** PI, phosphatidylinositol. Values shown are mean ± SD (*n* = 5). ^*^p < 0.05, ^**^p < 0.01, ^***^p < 0.001, two-sided Student’s *t*-test.

**Figure 5:**
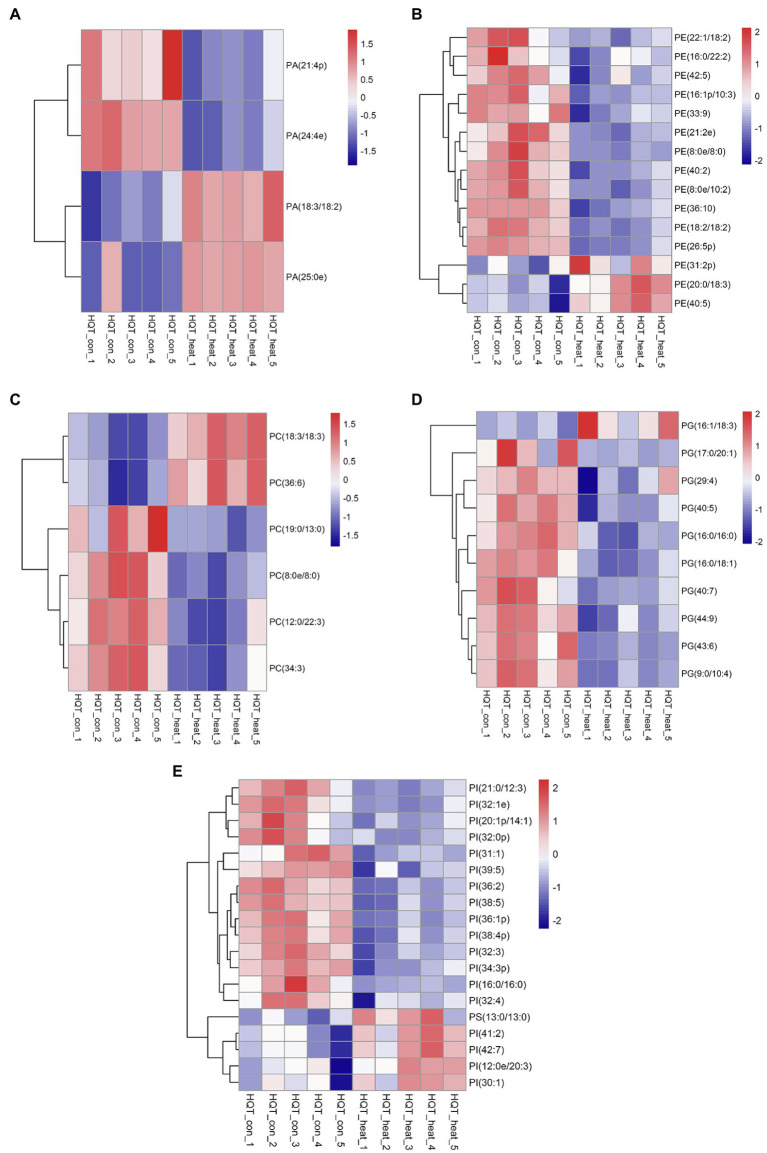
Heat map of profiles for the phospholipid species identified in leaves of *B. grandis* under heat stress. Each column represents an LC–MS measurement from five biological replicates of control condition (HQT_con_1 to HQT_con_5) or five biological replicates of heat treatment (HQT_heat_1 to HQT_heat_5). Fully extended leaves detached from 173-d-old plants of *B. grandis* were at control condition (22°C for 2 h) or challenged with heat stress (45°C for 2 h). The phospholipid species of **(A)** PA, phosphatidic acid, **(B)** PC, phosphatidylcholine, **(C)** PE, phosphatidylethanolamine, **(D)** PG, phosphatidylglycerol, **(E)** PI, phosphatidylinositol and PS, phosphatidylserine, measured from the control and heat treatment conditions were shown.

Phosphatidic acid (PA) has been identified as a new class of lipid mediators involved in regulating plant growth and developments, such as membrane tethering during cell development and structural effects on cell membranes (Zhang et al., 2004; Wang et al., 2006; Higashi and Saito, 2019). In addition to its structural roles, extensive studies have been focusing on PA serving as a lipid signaling molecule regulating the responses of plants to a variety of biotic and abiotic stresses, such as heat, chilling, drought, pathogen attacks, and hormonal signaling of abscisic acid (Zhang et al., 2004, 2019; Wang et al., 2006; Hou et al., 2016; Higashi and Saito, 2019; Li and Wang, 2019; Li et al., 2019b). In this study, heat stress resulted in a dramatic increase in the total amount of PA species in leaves of *B. grandis* in response to heat stress (45°C, 2 h; Figure 4A). This heat-induced accumulations were mainly attributed from the molecular species of PA, such as PA (18:3/18:2) and PA (25:0e) whereas the reduction in the levels of PA (21:4p) and PA (24:4e) was detected in response to heat stress (Figure 5A). Our results are in agreement with previous reports that heat stress induced a rapid accumulation of PA in leaves of Arabidopsis (Mishkind et al., 2009; Horvath et al., 2012; Higashi and Saito, 2019).

In heat-challenged leaves of *B. grandis*, 15 species of phosphatidylethanolamine (PE) were measured (Figure 5B). Heat stress also increased the total amount of PE (Figure 4B), and this increase was mainly driven by accumulation of three PE species including (31:2p), (20:0/18:3), and (40:5; Figure 5B). Inconsistent with our results, the total amount of PE was reduced in leaves of Arabidopsis (Higashi et al., 2015) and wheat (Narayanan et al., 2016b). In agreement with our results, heat stress led to a significant increase in the content of PE whereas heat treatment decreased the levels of 18:3-containing PE species (34:3-PE, 36:5-PE, and 36:6-PE) in turfgrasses and tall fescue (Su et al., 2009; Zhang et al., 2019). We also found that the levels of 18:2- and 18:3-containing PE species (36:4-PE and 36:10-PE) were reduced in response to heat stress (Figure 5B), which is consistent with the results reported in turfgrasses and tall fescue as mentioned above. With respect to the changes of phosphatidylserine (PS), heat stress-induced accumulations of PS were reported in a variety of plant species, such as wheat (Narayanan et al., 2016a), turf grasses (Zhang et al., 2019), and desert shrub (*A. lentiformis* of the *Chenopodiaceae* family; Li et al., 2015). In this study, the accumulation of PS was also measured in leaves of *B. grandis* in response to heat stress (45°C, 2 h; Figure 4C).

### Heat Stress Reduces the Levels of PC, PG, and PI

Phosphatidylcholine (PC) is one of the major structural components of cellular membranes and plays other critical roles in plant vegetative and reproductive developmental stages(Wang et al., 2006; Higashi and Saito, 2019;Nakamura, 2021; Yu et al., 2021). PC is required for cell proliferation and differentiation (Nakamura, 2021; Yu et al., 2021). Based on the previous studies, the content of the major phospholipids PC was changed in different patterns in a variety of plant species, which were not very consistent, probably due to dynamic turnover of the PC lipids and differences in plant sources and growth conditions (Nakamura, 2021; Yu et al., 2021). In this study, total amount of the PC species was found to decrease significantly in response to heat stress (45°C, 2 h) in leaves of *B. grandis* (Figure 4D). With respect to the detected 6 species of PC, 19:0/13:0-PC, 8:0e/8:0-PC, 12:0/22:3-PC, and 34:3-PC were found to decrease whereas the levels of 18:3-containing PC increased in response to heat stress (Figure 5C). In agreement with our findings, heat stress treatment was reported to increase the accumulations of 18:3-containing PC in leaves of Arabidopsis (Higashi et al., 2015; Mueller et al., 2017). Phosphatidylglycerol (PG) is mainly chloroplast-localized as one of the phosphorous-containing membrane lipids. As shown in Figure 4E, heat stress caused a significant reduction in total amount of the PG species in leaves of *B. grandis*. Ten of the PG species were measured, and the heat-induced reduction was mainly resulted from the molecular species of PG, such as 17:0/20:1-PG, 29:4-PG, 40:5-PG, 16:0/16:0-PG, 16:0/18:1-PG, 40:7-PG, 44:9-PG, 43:6-PG, and 9:0/10:4-PG whereas the level of 16:1/18:3-PG increased under heat stress (Figure 5D). Accumulated data suggest that the largest proportion of lipid species contain 36 and 34 acyl carbons, indicating two 18-C fatty acid chains or a combination of 16-C and 18-C chains when lipidomic analysis was performed in a variety of plant species (Devaiah et al., 2006; Okazaki and Saito, 2014; Higashi et al., 2015; Mueller et al., 2015, 2017; Yu et al., 2021). In contrast, the lipid species with 38 acyl carbons or more, containing one chain of 20–26 carbons (very long-chain fatty acids, VLCFAs) plus one 18-C chain are generally less abundant (Devaiah et al., 2006; Okazaki and Saito, 2014; Higashi et al., 2015; Mueller et al., 2015, 2017; Yu et al., 2021). As we found, the four decreasing species with VLCFAs are highly desaturated, such as 40:5-PG, 40:7-PG, 43:6-PG, and 44:9-PG, which may contain one chain of 20–26 carbons (Figure 5D). In general, a significant decrease was measured in the highly unsaturated PG species with VLCFAs, which could be involved in stabilizing membrane systems given the importance of desaturation levels of lipids in adjusting the membrane fluidity. It is worth noting that 36-C PG was not detected in this study, which is consistent with results reported previously in Arabidopsis (Welti et al., 2002; Devaiah et al., 2006). However, another report suggests that 36-C PG is present in Arabidopsis, albeit at low signal intensity(Higashi et al., 2015).

Phosphatidylinositols (PIs) are a class of cellular signaling lipids acting in cellular processes, including membrane trafficking, cytoskeleton organization, polar tip growth, and abiotic and biotic stress responses (Thole and Nielsen, 2008; Ischebeck et al., 2010; Hou et al., 2016; Meijer et al., 2017). As measured in leaves of *B. grandis* in response to heat stress (45°C, 2 h), the total amount of the PI species decreased dramatically in comparison with control condition (Figure 4F). There were 18 PI species detected with the heat-induced reduction in the levels of the PI species including 21:0/12:3-PI, 32:1e-PI, 20:1p/14:1-PI, 32:0p-PI, 31:1-PI, 39:5-PI, 36:2-PI, 38:5-PI, 36:1p-PI, 38:4p-PI, 32:3-PI, 34:3p-PI, 16:0/16:0-PI, and 32:4-PI and the levels of 41:2-PI, 42:7-PI, 30:1-PI, and 12:0e/20:3-PG increased under heat stress (Figure 5E). Based on the reported results, heat stress increased the total amount of the PI species in Arabidopsis (Higashi et al., 2015) and turf grasses(Zhang et al., 2019) whereas no significant difference was detected in the total amount of the PI species in wheat(Narayanan et al., 2016b). In comparison with the heat-induced accumulation patterns of the PI species, the plants of *B. grandis* showed specific patterns different from previous reports.

### Heat Stress Causes Reductions in the Levels of Lysophospholipids and Sphingolipids

**Figure 6:**
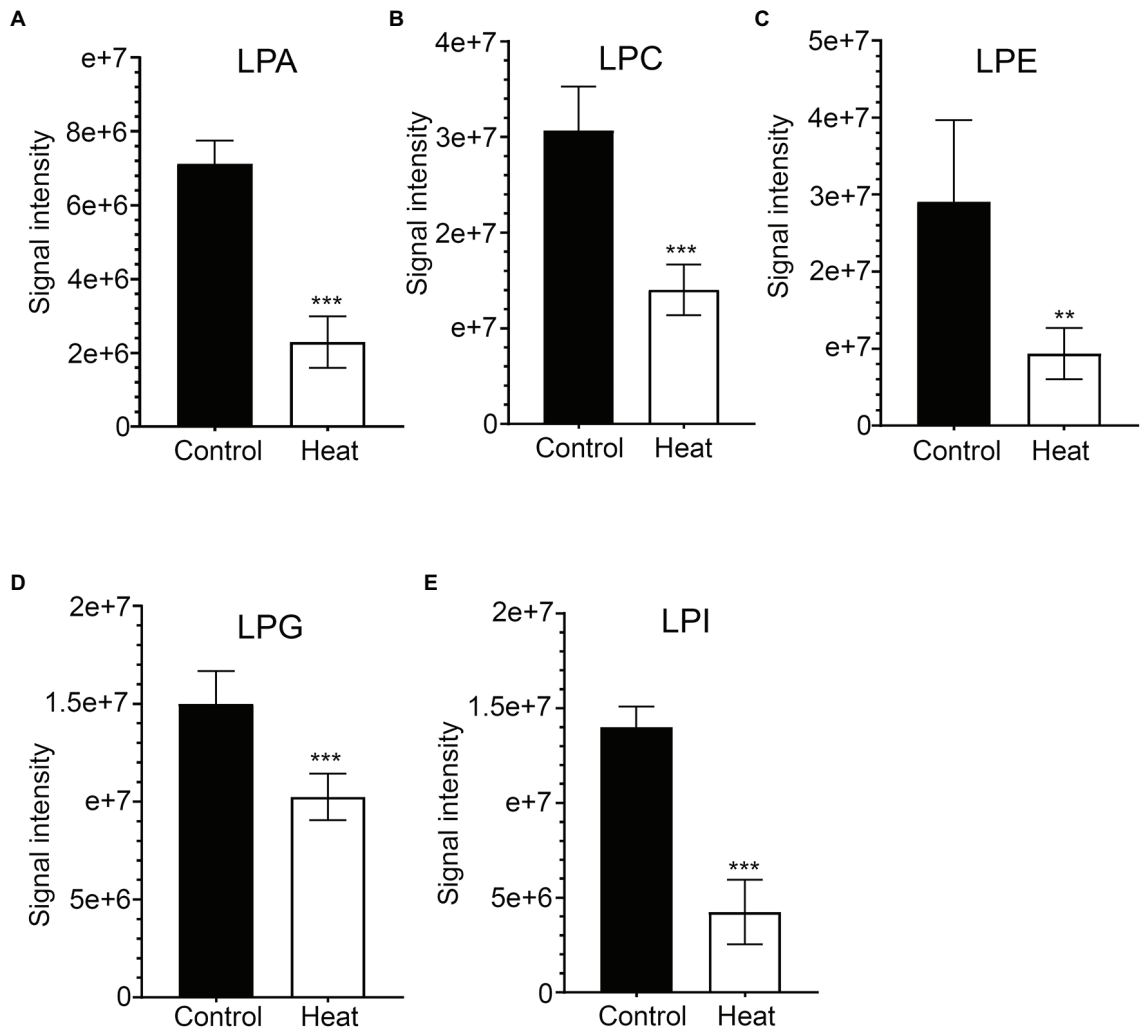
Changes in the levels of lysophospholipid molecular species in response to heat stress. Fully extended leaves detached from 173-d-old plants of *B. grandis* were at control condition (22°C for 2 h) or challenged with heat stress (45°C for 2 h). **(A)** LPA, lysophosphatidic acid; **(B)** LPC, lysophosphatidylcholine; **(C)** LPE, lysophosphatidylethanolamine; **(D)** LPG, lysophosphatidylglycerol; **(E)** LPI, phosphatidylinositol. Values shown are mean ± SD (*n* = 5). ^**^p < 0.01, ^***^p < 0.001, two-sided Student’s *t-*test.

As bioactive molecules, lysophospholipids (LysoPLs) are usually the hydrolysis products of glycerophospholipids catalyzed by phospholipases at the sn-1 or sn-2 position of the glycerol backbone. The most common LysoPLs usually include lysophosphatidic acid (lysoPA), lysophosphatidylcholine (lysoPC), lysophosphatidylethanolamine (lysoPE), lysophosphatidylglycerol (lysoPG), and lysophosphatidylinositol (lysoPI; Higashi et al., 2015; Hou et al., 2016). Accumulation of LysoPLs was reported in a variety of plant species when challenged with abiotic and biotic stresses, such as freezing, wounding, pathogen infection, or the application of elicitors (Lee et al., 1997; Narvaez-Vasquez et al., 1999; Scherer, 2002; Viehweger et al., 2002; Welti et al., 2002; Wi et al., 2014). Under heat stress, no significant changes were detected in the contents of three lysophospholipid classes (lysoPC, lysoPE, and lysoPG) in two representative species of alpine scree plants (*Saussurea medusa* and *Solms-Laubachia linearifolia*; Zheng et al., 2011). In this study, total amounts of the most common LysoPLs species were detected to decrease significantly in leaves of *B. grandis* in response to heat stress (45°C, 2 h; Figures 6A–E). These LysoPLs species include two species of lysoPA (16:0 and 18:2), five species of lysoPC (16:0, 18:1, 18:2, 18:3, and 20:5), two species of lysoPE (16:0 and 18:2), three species of lysoPG (16:0, 16:1, and 18:2), and two species of lysoPI (14:0e and 16:0; Figure 7). LysoPLs are thought to be marker molecules representing instant responses to a variety of stress conditions given that they are usually the hydrolysis products of glycerophospholipids catalyzed by phospholipases in a variety of plant species when challenged with abiotic and biotic stresses(Wi et al., 2014; Higashi et al., 2015; Higashi and Saito, 2019).

**Figure 7:**
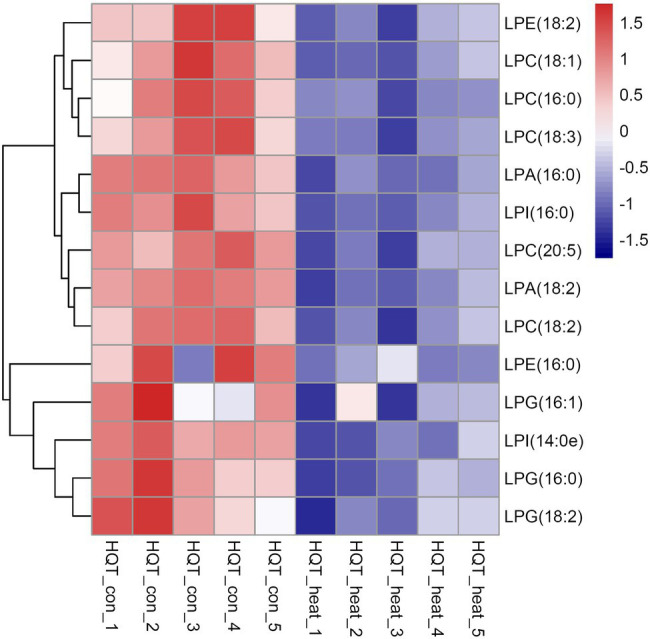
Heat map of the significantly differential lysophospholipids in leaves of *B. grandis* in response to heat stress. Fully extended leaves detached from 173-d-old plants of *B. grandis* were at control condition (22°C for 2 h) or challenged with heat stress (45°C for 2 h). Each column represents an LC–MS measurement from five biological replicates of control condition (HQT_con_1 to HQT_con_5) or five biological replicates of heat treatment (HQT_heat_1 to HQT_heat_5). The lysophospholipid species of lysophosphatidic acid (LPA), lysophosphatidylcholine (LPC), lysophosphatidylethanolamine (LPE), lysophosphatidylglycerol (LPG), and phosphatidylinositol (LPI) measured from the control and heat treatment conditions were shown.

**Figure 8:**
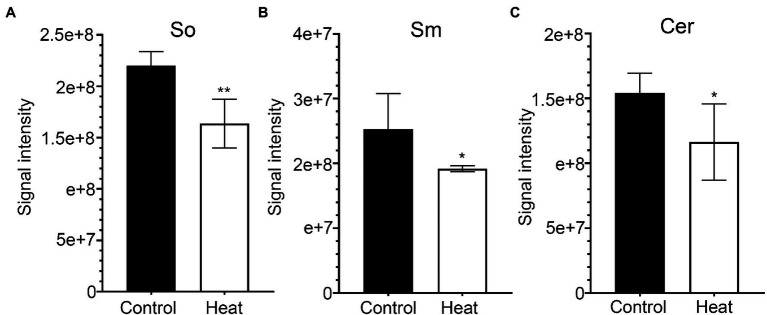
Changes in the levels of sphingolipid molecular species in response to heat stress. Fully extended leaves detached from 173-d-old plants of *B. grandis* were at control condition (22°C for 2 h) or challenged with heat stress (45°C for 2 h). **(A)** So, sphingosine; **(B)** Sm, sphingomyelin; **(C)** Cer, Ceramides. Values shown are mean ± SD (*n* = 5). ^*^p < 0.05, ^**^p < 0.01, two-sided Student’s *t-*test.

Sphingolipids constitute up to 40% of those lipids making the plasma membrane (PM), forming a significant portion of the lipids present in higher plants (Cacas et al., 2016). In plants, sphingolipids are thought to be major components of plasma membrane, tonoplast, and endomembranes (Markham et al., 2013). It is generally accepted that sphingolipids accumulate in the outer leaflet of the PM and are basically characterized by a ceramide formed by a sphingoid base amide linked to an acyl chain (Markham et al., 2013; Carmona-Salazar et al., 2021). In this study, three molecular species of sphingolipid-related metabolites (So, sphingosine; Sm, sphingomyelin; Cer, ceramide) were detected in leaves of *B. grandis* and the total amounts of three molecular species were significantly reduced in response to heat stress (45°C, 2 h) in comparison with control condition (Figures 8A–C). Extensive studies suggest sphingolipid-related metabolites as potential signaling molecules in mammalian cells as well as in some studies with yeast. Previous work showed that sphingolipid-deficient strains of *Saccharomyces cerevisiae* are heat-sensitive, suggesting that sphingolipids are necessary for surviving heat stress(Wells et al., 1998). It was reported that sphingolipid long-chain bases act as signaling molecules that regulate growth and responses to heat stress in *S. cerevisiae* (Dickson et al., 2006). In yeast, phytosphingosine, thought to be a putative sphingolipid second messenger, acts as a mediator in regulation of heat stress signaling and activation of ubiquitin-dependent proteolysis *via* the endocytosis vacuolar degradation and 26 S proteasome pathways (Chung et al., 2000). Based on the accumulated findings, sphingolipids function as bioactive signals and play critical roles in a variety of physiological processes and environmental responses, including programmed cell death (Liang et al., 2003; Shi et al., 2007; Simanshu et al., 2014), pathogen-induced hypersensitive response (HR; Liang et al., 2003; Wang et al., 2008; Gan et al., 2009), ABA-dependent guard cell closure (Ng et al., 2001; Coursol et al., 2003; Worrall et al., 2008), host–pathogen interactions (Takahashi et al., 2009; Peer et al., 2010; Rivas-San Vicente et al., 2010; Sanchez-Rangel et al., 2015), and low-temperature stress responses (Cantrel et al., 2011; Dutilleul et al., 2012). According to the studies in yeast, sphingolipids could act as signaling molecules functioning in the physiological adaptations to heat stress in plants. In higher plants, how sphingolipids affect heat stress signaling and thermotolerance has remained unexplored (Markham et al., 2013; Batsale et al., 2021).

## Conclusion

Generally, lipids as determinant factors regulate membrane fluidity and stability in keeping the structure and function of plant cells based on the lipid composition and fatty acid unsaturation levels under normal and stress conditions. In this study, the analysis of lipidomic remodeling suggests that the content and composition of membrane lipids in leaves of Begonia (*B. grandis*) were dynamically changed under heat stress. Particularly, heat stress induces a very dynamic remodeling process in the level and composition of glycerolipids, the major constituents of membranes, suggesting that the glycerolipid remodeling in response to heat stress is a marked process with keeping the integrity and optimal fluidity of the membrane systems in Begonia plants. One of the distinct characteristics of membrane composition during alterations under heat stress is the substantial increase in the type of TG (18:3/18:3/18:3), marked the first stage of adaptation processes. The second distinct characteristic of membrane composition in leaves of Begonia plants is that heat stress induces dramatic reductions in the total amounts of lysoPLs, which is an interesting lipid remodeling pattern for Begonia plants given that lysoPLs are thought to be a sensitive indicator for interpreting the strength of stress responses in higher plants. The elucidation of the specific lipid remodeling patterns would be the focus in our future studies.

## Data Availability Statement

The original contributions presented in the study are included in the article/supplementary files, further inquiries can be directed to the corresponding authors.

## Author Contributions

F-QG and YY conceived the project and provided supervision. F-QG, A-ZS, L-SC, MT, and J-HC designed experiments. A-ZS, L-SC, MT, and J-HC performed experiments. J-HC, A-ZS, and L-SC analyzed the data. F-QG, YY, A-ZS, L-SC, MT, and J-HC wrote the manuscript. All authors contributed to the article and approved the submitted version.

## Funding

This work was supported by grants from the National Natural Science Foundation of China (U1812401, 31770314, 32000211, and 31600225), the Ministry of Science and Technology of China (National Key R&D Program of China, 2020YFA0907604), and the Chinese Academy of Sciences (The Strategic Priority Research Program, XDB27040105).

## Conflict of Interest

The authors declare that the research was conducted in the absence of any commercial or financial relationships that could be construed as a potential conflict of interest.

## Publisher’s Note

All claims expressed in this article are solely those of the authors and do not necessarily represent those of their affiliated organizations, or those of the publisher, the editors and the reviewers. Any product that may be evaluated in this article, or claim that may be made by its manufacturer, is not guaranteed or endorsed by the publisher.
